# Psychotherapy in Habitual Constipation

**Published:** 1911-06

**Authors:** E. T. Gibbs

**Affiliations:** Gainesville, Ga.


### PSYCHOTHERAPY IN HABITUAL CONSTIPATION.

E. T. Gibbs, M.D., Gainesville, Ga.

In reporting this series of cases, relieved of constipation by pschotherapy, no originality is claimed for the idea, as it was gotten from Du Bois (1), of Berne, who has used the method successfully for over thirty years. Lyon (2), in "The Radical Cure of Habitual Constipation" also mentions Du Bois. Davis merely mentioned *hypnotic suggestion* for the same purpose in Sajous Analytical Cyclopedia of Practical Medicine, only alluding to it, meriting as yet (1904) but little confidence.

Inasmuch as there is considerable disagreement as to the definition of the word psychotherapy, I wish to present and discuss the method followed in the treatment of these cases.

Some look upon psychotherapy as synonymous with hypnotism or suggestion in the waking state; some think it only applicable to psychic disorders; while still others believe the entire phenomena purely psychic.

Some of the religious cults, while beginning with cases diagnosed by medical men, as so-called functional diseases, have now become bold enough to proceed without aid from the medical man. Probably by a combination of suggestion, persuasion and hypnotism, they became so enthused over certain magical results, that they combined the whole with religion and looked upon the cures as being of divine origin. Indeed, with some of these cults, even the existence of disease is denied, and only an unqualified acceptance of their faith is necessary for freedom from any malady.

On the other hand, psychotherapy has been practiced by all physicians, we might say, since medical history began. Suggestion alone, suggestion by drugs, and in various other ways. Hypnotism for a while was highly lauded, but of recent years it is being largely abandoned and psychotherapy, as it is now consciously used, instituted in its stead. Even those men who claim to see harmful effects from hypnotic treatment, are willing to admit that a clearing up of psychotherapy has been due to the exhaustive study of hypnotism by men within the medical profession. It was Berheim who pointed out that hypnosis within itself is nothing, for it is merely suggestion while in the walking state, which puts the subject into the so-called trance, thus allowing himself to become a slave to the wishes of the suggester. Inasmuch as pure ideas or sensations cannot exist independent of physical conditions in the cerebrum, which is the claim of physiologists, Barker (4), has suggested, to avoid confusion with spiritualism or other mystical conceptions, that it might be better to speak of psychophysical influence and psychophysical therapy, than to use the terms in vogue. For if the above assumption be true, he points out that any influence which exerts a psyshic effect also produces a physical change in the body, so it is then obvious that physical and chemical influence which lead to alterations in the structure and function of the neurones of the pallium may have a profound effect on the psyche.

The number of patients treated and relieved, is one hundred and six (106), without a failure, if permission is granted to shift two cases to the column for errors in diagnosis. These were the only ones abandoned in the series and will be referred to later. I especially desire to emphasize the fact that the method was only applied to those of the habitual type, so it will be unnecessary to discuss the various causes of constipation, such as obstruction, fissures, hemorrhoids, ulcers, extreme atony or the type associated with various diseases where thorough depletion is frequently desirable or even necessary. Cases with dilatation of the colon, where we get at times distention of the whole or various parts of the colon, accompanied by such symptoms as headache, vertigo, nausea, gaseous eructations, slight dyspnoea (generally at night), pains in the back and at times various nervous symptoms, were not classed as unfavorable subjects; there being a number of such cases in the series. This class were small eaters, passed urine frequently but the twenty-four hour specimen usually showed a deficient quantity, highly colored, most always a marked indican reaction and occasionally highly charged with phosphates. These were kept under observation until they could be established upon a liberal and suitable diet, but if medicine was to be given for the condition, it was withheld until a few days after the bowels had begun functioning. It seemed to me that the establishment of regular peristaltic action, a proper diet and a re-education of their ideas concerning themselves, has largely to do with their future well being. Am sorry I failed to keep a record of the cases thought to be unsuitable for the treatment. However, there were several anal fissures, one fistula in ano, hemorrhoids (severe), retroverted uteri, one rectocele and four morphine habitues in the group. The latter were accused and hard pressed before histories were obtained of morphinism. One case was thought clinically to be obstruction of bowel: he has since died. Three of the surgical conditions were operated upon and afterward given psychotherapeutic directions for relief of constipation. The unfavorable cases of the habitual type, so far as permanency of cure is concerned, will be referred to later.

As to causes of habitual constipation, I believe the name largely explains the condition, but the numerous contributing factors come in for their share of credit. Among which diet, both the small eater and those who eat largely of foods which furnish only a small amount of nondigestible food and debris. Frequently, people lay the cause of the trouble to sedentary habits, which, no doubt, play a part but, on the other hand, it is not unusual by any means, to find them among laborers, walkers, athletes, and so on. Among people who have demands upon their time at all hours, as various professional and business men, the busy housewife, and those who do not select and observe a regular hour, is where we find the bulk of cases. Then comes the conviction of inability or almost complete dependence on some other medical agent rather than upon natural stimulations.

Just as every disease, whether it be physical or psychic, should be individualized, so will every case of constipation have to be dealt with somewhat differently.

In these cases, an effort has been made to use only rational persuasion. It will be evident that suggestion comes in frequently, but particular pains are taken to make no statement that is not what we believe to be absolutely true from a study of both physiology and psychology. Then it will be possible to convince or persuade the patient of the logic of each statement, step by step.

A diagnosis of the habitual type having been made, in a conversation, the patient's ideas are studied, and we find, I might say, valuable obstacles which our persuasion is to overcome. As we are going to teach the patient self-reliance, it will be necessary to get his full consent and also to inspire him with determination to enter upon a plan of treatment that is independent of drugs. He can be questioned upon the various remedies from which relief has been sought, and when you ask why he jumps from one remedy to another, the answer will be, because the remedy fails after a time. He will then easily see that relief is not near at hand by the method he is now pursuing, and if his attention has been thoroughly grasped, he at once becomes interested provided the plan appears plausible to him. The question of headache, in case of a lapse, is often a serious obstacle, and as we cannot say that the constipation is free of responsibility, merely add "No risk, no gain," thus persuading him to be content in the presence of a little pain for a short while, should it become necessary.

The influence of habit in our lives generally is stressed, and a few examples suitable for the particular patient, are cited. It will be easy for the patient to recognize that if the regular retiring hour has been ten o'clock, the eyelids will usually become heavy at that time. On the other hand, where a regular hour of retiring at twelve has been well established, it will be in vain to sleep before this time, should he go to bed at nine, even though he may have passed quite a strenuous day. Just so, we have noticed our parents or grand-parents arising at six in the morning, when we have felt that they would do well to take more repose, but the habit has been formed and they persist, even though they may lie down for a nap before the noon hour has arrived. The patient will usually cite other examples to support your statement, thus showing he has caught the idea. These are facts, very likely of which he is already cognizant; but in matters of which one feels sure, it is quite a relief to confide in a dear friend and receive his encouragement. Just so, the patient has not analyzed these facts, and applied the comparisons which we suggest. When they think of the regularity of habit in those not constipated, they will see no reason why they themselves should not establish the habit. The human organism may be compared to an imposing locomotive engine, and reference made to the necessity of having an engineer and fireman to operate the latter. Just so, when we are constantly devising means for the operation of our bowels, they are accustomed to aid, so invariably wait for it. On the other hand, our bodies, when in a state of health, have been equipped as self-oiling and self-regulating pieces of machinery, and a good way to teach one self-reliance is to place him on his own responsibility. The functions of digestion are gone over in a very general and simple way, of course, when opportunity is taken to lay particular stress upon peristalsis, mastication, assistance of saliva as a lubricator, deglution, journey of food to the stomach, and on through the intestinal canal. Fact that in upper end of small intestine, the nutritious portion of food is absorbed into the system through tiny blood vessels lining their walls, the remaining indigestible, undigested food with debris go to be thrown off as waste. Some simple illustrations is used to illustrate peristaltic movements, the means by which food is carried along its course. One who has seen a field of wheat just before harvest time can recall a mental picture of the beautiful ripple which passes over the field under the influence of a gentle breeze, or how slight continuous strokes on a sheet of still water will send wave after wave, each chasing the other. These can be imagined as similar to the peristaltic waves which are set in motion by the complex stimulations. Epsom salts act as a purgative by reason of its abstraction of water from the intestinal blood vessels, because it is not readily absorbed, thus directly it stimulates peristalsis. Such stimulations may be cultivated to act in an automatic way at regular intervals, provided we indulge persistence. After the habit is formed, one stimulation will be all that is required, but at first we must summons a number of continuous invitations.

No directions, very much out of the ordinary, are given, such as exercise, special diet, water at stated intervals and so on, for many of the patients would neglect some particular and would at once realize that they had not followed directions; consequently, the object sought for would not be confidently expected. Find out the usual routine of arising, breakfast time and hour of departure for work, making nothing arbitrary except a promise of regularity in execution of these three acts until the habit is well established, when he can then rely on the stimulation coming at a certain number of minutes following the breakfast hour, but this notice must continue to be regarded as an important duty to be performed in his daily routine.

The act of getting from bed, movements of body in dressing, and preparing one's toilet may be enumerated as three successive invitations, a glass of cold water is now taken for the fourth, then comes breakfast for the fifth, and the act of going to stool is considered the sixth. Coffee, dry toast and crisp bacon may be ordered for breakfast, with any additional food patient may desire. The meal will keep in action the previous stimulations already begun. Say the hour for stool has been set for thirty, forty-five or sixty minutes following beginning of meal, according to amount of time the patient usually has before his hour for business. Directions are now given as to the use and abuse of abdominal muscles and the will power. Defecation is stimulated by entrance of the mass into the rectum, which sets up the peristaltic wave, but only a slight and gentle contraction of abdominal muscles should be induced at the beginning. The sphincter which acts as on inhibitor, will often contract all the tighter if the muscles and will are brought to act too violently at an inopportune moment. The fecal column may also possibly be deflected from its proper course. Some patients state that they simply wait for the desire, and then offer gentle assistance. This ends the treatment, and in people other than neurasthenics or people without persistence or determination, is usually enough to accomplish the result. In such instances, it will be necessary to keep up encouragement for a few days. I do not, unless hard pressed, tell the patient that an enema will be given on third day, but simply wait until the time arrives, then if necessary the usual S. & W. enema is ordered, a quart introduced, in the knee-chest position, so it can course well into the colon to be retained for fifteen minutes by the clock if possible.

It is not unusual to have the patient state, "But doctor, I have many times tried just such a plan, but invariably with the same result, failure." "Very well," I add, "One or two failures should not absolutely guarantee another; and, remember, we are going to summons at this time a number of successive stimulations. A barrel may hold many quarts of water, but if one keeps pouring quarts into it, finally this one cup will cause the overflow." Remind him here that the method has succeeded with others, if it has, and simply urge a trial. I might add here, that I believe a certain amount of enthusiasm, together with sincerity in expectation of outcome, is a valuable, if not a *necessary* adjunct to the physician giving the directions.

As stated, the number of cases treated is one hundred and six (106) without a failure. Three enemae were given but all to the same patient, who will be referred to later. It seemed as though the cases were not really constipated but merely had the conviction of constipation. However, no case is reported except those who gave histories of protracted constipation, some having had trouble with it since childhood, in an obstinate form.

Only two cases were given directions which had to be abandoned on account of mistaken diagnosis. These were seen at Dispensary of Baltimore College of Physicians and Surgeons. They refused vaginal and rectal examinations but gave such good histories of habital constipation that they were undertaken. One was suffering with marked gastropoasis and floating kidneys, the other had a general enteroptocis. Both markedly neurasthenic but were fairly well nourished, and in seemingly good physical condition. On the third day enemae were ordered, and histories given of rather profuse hemorrhage, marked pain, but little result from the injections. One submitted to examination by a gynecologist, retroversion of uterus, pressing upon the rectum and internal hemorrhoids were found; she was advised to go into the hospital for operation, which she did. My directions were, of course, discontinued. They would have been numbers seventy-three and four respectively in the series. I was anxious to see the results in such cases but eagerness caused error in diagnosis. They stated later that blood in stool was denied at first to escape examination.

Ages of patients ranged from five to sixty-eight years.

Women predominated about three to one.

Time of initial conversation ranged from thirty minutes to one hour and a half.

Both highly intelligent and ignorant persons responded alike. No subject would, of course, be undertaken who did not have necessary intelligence to follow and understand the outlined directions.

As to relapses, I am unable to give very definite data, but have observed very few. I lay not a good deal of stress on this however, for the following reasons, except in those suffering from neurasthenia or some nervous malady. It would be expected that these have a tendency to relapse, because they do not usually have any surplus will power, are easily discouraged and not systematic generally. Their appetites are also capricious, and nearly always applying a cause for every result, many would get the conviction that they should be constipated, because they had thought the constipation responsible for their whole train of symptoms, and of course these, except very occasionally, would not be expected to leave on so short a treatment. I find the method an excellent one in such cases where we are going to proceed with a treatment looking to the cure of the patient. For when the bowels move in a few days by such directions, it is a decided encouragement to the patient from the beginning, and helps wonderfully in establishing confidence in themselves and also the physician, both of which are so necessary for a speedy recovery, no matter what line of treatment is to be followed. As before stated, the suggestion involved is made rational so far as possible; consequently, the patient is able to see just how he was cured, and should be able to remain so by the same means, provided he does not become careless. In this case I think anyone who has not been a sufferer is liable to the same thing should he become negligent. Had one old gentleman fifty-six years old who skipped one day, and then took salts, and continued thereafter for a few days When he mentioned the relapse to me, I simply said in about so many words, "Leave off the salts and apply the original remedy, but make up your mind right now, to depend on yourself and not the salts." He was relieved the second morning following. On account of just such an occurrence as this, I always emphasize the fact that it may become necessary to resort to a flushing of the bowels, from directions of a physician, for example, but that it will be no trouble to resume the habit if they will only apply the original directions, which they will naturally come to know so well.

It is not necessary for the patient to have strong confidence as the following illustration will show. A doctor of middle age, an active practitioner, had been suffering from obstinate constipation for eight or nine years. Bowels had been regular previous to this time. He attributed the cause to continuous driving, having gotten into the habit on account of irregular hours of arising, breakfasting, and frequently being called out while yet at breakfast. It was with some reluctance that I undertook the case, remarking at the time in the presence of three physicians, that I had never given a physician this treatment, but had an idea that they would be hard subjects; however, you may yield, as my directions will at least have the advantage of demanding no disagreeable medicine. An unusual number of oppositions had to be overcome, most serious of which was the headache which would follow, if he did not have two movements daily, which he at least made an effort to have. He was in Baltimore at the time and remarked that he would try it when he returned to his home. "Your confidence must be rather slim, doctor, from the doubt implied," I ventured. He replied, "Not only shaky, but I have absolutely no faith in it whatever, for the simple reason that I have tried just such means many times." The subject of headaches, which he thought to be due to auto-intoxication was again gone over. From his history, physical condition and so on, I did not believe the auto-intoxication was much of an element, if any. He gave a history of the regular old-fashioned sick headache, from which he had suffered before the constipation came on, and, in fact, had been troubled less of recent years than formerly. I urged a trial, insisted upon one movement daily, and cautioned him simply not to allow but the morning action, if he could possible prevent it. "Burn the bridges behind you and put up with a little head-ache if it should come. Do not be afraid of a little pain, when I believe you can be relieved, adding, in a joking way, that he could be relieved of both the inconvenience and expense of resorting to Dr. Hinkle's pink pills." He gave his promise and had a normal passage the following morning and continued to do so as long as I had him under observation.

An intelligent woman gave history of obstinate constipation since childhood, this having always been a source of worry to her mother. Did not remember ever having gone without a laxative for longer than five days. At present, she was alternating between senna tea and sal hepatica. She was relieved over night, after an hour's conversation.

Will cite one case that responded promptly while on a rest cure. A woman of thirty-eight, hystero-neurasthenia of long standing, was put to bed and restricted to milk diet. Her symtoms were numerous and complex. Weakness, amounting to almost continual exhaustion, occipital headaches, pain in back, palpitation, precordial pain that had prevented lying on left side for some years, simultaneous swelling of abdomen and dyspnoe, especially at night, fear of impending death and so on. Physical examination complete and negative; was also fortunate in getting a history from a very competent physician who had formerly treated her. Enema was ordered on third day following convarsation (on bowels) and was notified that two had been given in knee-chest position but without results. Examination made and impacted feces discovered. It had been two weeks since vaginal and rectal examination had been made, and at that time the mass was not present. With the aid of a stiff rectal tube and an enema of glycerin and water, the mass was fairly easily dislodged. Bowels moved third day following, the same day on which bread and butter were added to the milk diet. Treatment was continued for two months along psychotherapeutic lines, the symptoms all disappearing with a seemingly good recovery. As to the bowels, the patient has since passed through both typhoid and malarial fever, and measles, during which she received calomel, epsom salts and enemae, but quickly resumed normal movements following the illness, without any further directions. First treated in June and July, 1910.

The time of recovery in all cases being within six days, brings up an interesting psycho-physiological problem. Howell's physiology sums up the act as follows: The whole act seems to be an involuntary reflex. The physiological center for the movement probably lies in lumbar porton of cord; and has sensory and motor connections with rectum and muscles of defecation; but the center is probably provided with connection with centers in cerebrum, through which the act may be controlled by voluntary impulses and by various physical states. The effect of emotion upon defecation being a matter of common knowledge.

As to the establishment of the function: A liberal diet, especially of vegetables and fruits, free drinking water, regularity of chosen hour are all important. Then the entrance of feces into the rectum, setting up peristalsis in the lower portion and probably also throughout the whole intestinal canal, the play of the muscles, the reflex act from the supposed ano spinal center in the lumbar portion of the cord; all of these factors are important, but of the directions in this series, none of the physical means have had time to act when we see the bowels respond frequently on the following day. The diet has had no time to produce a change, nor has habit had time to become formed. It is quite evident that the psychic plays an important role. I cannot say how many cases would be relieved in so short a time or what proportion would resist such treatment altogether, for some undoubtedly would, but it seems that a suppression of the conviction certainly plays an important role.

I have just recently seen a case that beautifully illustrates a very sudden stimulation of the bowel movement and at the same time shows what a marked effect the will plays as in inhibitor. The subject was a patient of Dr. A. C. Harrison's at the Mercy Hospital, Baltimore, convalescing from an operation for abscess of the liver. It is true that he had suffered for three years with a moderate diarrhoea, but as he lies in bed, he will not dare sip his coffee without first getting on the bed pan. He knows absolutely in advance, that his bowels will move at once upon the taking of a hot drink. Before being confined to bed, the action was invariably the same, but if he were in a hotel, taking coffee with his meal, he could wait until the meal was finished, but would then always have to go in search of the toilet. But we cannot imagine that he was always the same time in getting there, so it is seen from the fact that never could he desist for a minute longer, what a marked effect both the conviction and will play.

Another patient, and not one who could be styled a neurasthenic, told me that she could not take a fresh drink of well water without becoming nauseated. A few minutes' talk was enough to dispel the nausea and it could have been done by hypnotism, suggestion or rank deception, but in the case where the conviction was suppressed by a rational persuasion, it seems that the patient would be less likely to relapse. I mention this instance merely to call attention to a conviction. At any rate, the psychology of defecation is a complex problem and I have been unable to find what seems to be a thorough explanation of the phenomena.

This method, I believe would be applicable to a majority of habitual constipationsits, but the time required for a diagnosis and the conversation is a draw-back for the method with busy practitioners. Whether as much time as I have allotted each patent is necessary I cannot say. Neither do I know how many patients would recover with simply a two or three minutes' talk on general directions, but the method for me was not very successful, and I was often told of failures by patients in this series. It seems, however, as though is should succeed with those who have the necessary persistence.

1. Du Bois, Paul.—The Psychic Treatment of Nervous Diseases, Funk & Wagnals Co., 1909.

2. Lyon, I. P.—The Radical Cure of Habitual Constipation. Trans. Assoc. Amer. Phys. 1908, xxiii, 482-493.

3. Davis, Nathan S.—Constipation, Sojous Analytical Cyclopedia of Practical Medicine. 1904, ii, 309-319.

4. Barker, Lewellys S.—Psychotherapy, Journal Amer. Med. Assoc., August 1, 1908. LI 368-371.

